# Is Capacity Building Training Effective for Changing Attitudes toward Health Inequalities? Experience from a Norway Grants Project in Lithuania

**DOI:** 10.3390/medicina55020052

**Published:** 2019-02-15

**Authors:** Mindaugas Stankūnas, Snieguolė Kaselienė, Akvilė Girčienė, Agis Tsouros, Mark Avery

**Affiliations:** 1 Department of Health Management, Faculty of Public Health, Lithuanian University of Health Sciences, Kaunas 44307, Lithuania; snieguole.kaseliene@lsmuni.lt (S.K.); miliakvile@gmail.com (A.G.); 2 Centre for Public Policy and Health, Durham University, Durham DH1 3LE, UK; tsouros@gmail.com; 3 Griffith University, Gold Coast 4222, Australia; mark.avery@griffith.edu.au

**Keywords:** health inequalities, capacity building, health policy development

## Abstract

*Background and Objectives*: In 2014–2017, the Lithuanian University of Health Sciences and partners implemented the project, ‘Development of the Model for the Strengthening of the Capacities to Identify and Reduce Health Inequalities’, which was financed by The Norwegian Financial Mechanism 2009–2014 Public Health Initiatives Program. One of objectives of this project was to increase the awareness about public health and related specialist knowledge and skills in the field of health inequalities. This paper evaluates the effectiveness of capacity-training sessions on capacity building regarding increasing the awareness and knowledge that is needed for addressing health inequalities. *Materials and Methods*: Participants attending capacity-building seminars were asked to complete the same questionnaires before and after these training sessions. A total of 145 questionnaires were received (response rate 71.8%). The evaluation of changes in the pre-survey and post-survey responses in relation to a nonparametric analysis of two related samples was performed using the Wilcoxon test. *Results*: Respondents were asked to identify the general importance of health inequalities to the national public health agenda. The pre-training median of the survey was nine (minimum four; maximum 10), and post-training was 10 [minimum five; maximum 10] (*p* < 0.001). Unemployed, low-paid, and low-educated people were identified as the most vulnerable groups of society in terms of health inequalities. A more effective tobacco and alcohol control was identified as the most important inequality measure needed. An absolute majority of participants emphasized the need for intersectoral collaboration for the effective reduction of health inequalities. *Conclusion*: The findings from our study suggest that capacity-building sessions can be effective measures for increasing awareness of health inequalities. It is expected that the outcomes of these training opportunities will act as facilitators for further engagement and ongoing approaches to addressing health inequalities.

## 1. Introduction

Socioeconomic health inequalities are one of the main challenges for health systems [1]. Previous studies have reported that Lithuania has the highest levels of health inequalities in the European Union and beyond [2]. Lithuanians face significant inequalities in mortality, self-reported health, lifestyle factors, and accessibility to health care and medications [3,4,5,6,7]. Therefore, the tackling of health inequalities was identified as a strategic goal in the Lithuanian Health Strategy for 2014–2025, which was approved by Lithuanian Parliament [8]. The inclusion of this goal in the main national health policy document illustrates the major political and public concerns relating to this issue.

There are optimistic expectations for positive improvement trends in tackling health inequalities in Lithuania. In 2014–2017, Lithuania implemented the project ‘Development of the Model for the Strengthening of the Capacities to Identify and Reduce Health Inequalities’. This project was financed by the Norwegian Financial Mechanism 2009–2014 Program ‘Public Health Initiatives’, and managed by the Lithuanian University of Health Sciences, Vilnius University, Klaipeda University, and the Institute of Hygiene. The project is aimed at the development of an evidence-based platform for health and health care inequalities monitoring, and strengthening the administrative capacities of people involved in policy making at national and municipal levels.

The project commenced (January–March 2015) with a national survey that aimed to evaluate the existing situation with respect to health inequalities monitoring and inequality reduction. It covered institutions that are related to tackling health inequalities at national and local levels. Study results have revealed that common obstacles and pitfalls for addressing health inequalities are related to: the lack of credibility of statistical data; a lack of uniform attitude toward health inequalities; the absence of practical guidelines; and a lack of inter-institutional collaboration [9,10]. These results suggested directions for further actions in conducting the project, such as the development of monitoring systems, and the preparation of recommendations for reduction and capacity building for public health professionals. The first two activities and project outcomes have been published separately [11], and this paper focuses on the component of the project related to capacity building. 

One of the key objectives of the project was to increase awareness and provide the skills that are necessary for monitoring and reducing health inequalities. Therefore, the project concluded by running capacity-building seminars for municipal and national-level public health specialists and policy makers. The aim of this paper is to evaluate and better understand the effectiveness of capacity-building training for increasing the awareness and knowledge that is needed for tackling health inequalities. To the best of our knowledge, there are no similar studies published elsewhere.

## 2. Materials and Methods

A quasi-experimental design [12] study for this project commenced between January–April 2017. The study participants consisted of people attending capacity-building training titled, *“Tackling of inequalities in health and health care: situation, challenges, and possibilities”* (*N* = 202). Participants at these training sessions represented different institutions and were involved in the monitoring and tackling of health inequalities in Lithuania. Training participants included (but were not limited to) representatives from the Ministry of Health of Lithuania, National Public Health Centre, National Sickness Fund, State Mental Health Centre, Police Department, and administrations of municipalities and local public health bureaus.

The capacity-building training sessions covered the most important topics related to the tackling of health inequalities in Lithuania. The list of learning and development topics is presented in Table 1. These topics were selected based on the outcomes and conclusions from other engagement activities in the project [10,11]. In total, nine sessions were delivered for participants (four for specialists of municipal public health bureaus, as well as five for health policy makers and partners from collaborating institutions). The curriculum was similar for both groups of participants. However, some specific emphasis was made for groups. The group of tutors was the same during the entire period of the capacity-building sessions (consistency), and problem-based learning elements were used. Tutors represented the four major public health training and research institutions: the Lithuanian University of Health Sciences, Vilnius University, Klaipėda University and the Institute of Hygiene. All of the training was run in a conference center in Kaunas (Lithuania).

**Figure 1:**
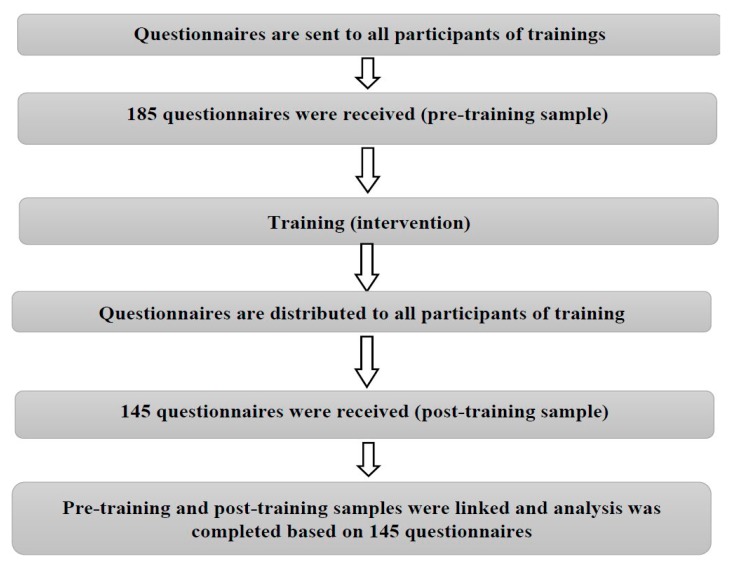
The scheme of the study.

All of the invited participants at these trainings sessions were asked to complete a pre-training questionnaire and send them back to the study investigators. We received 185 questionnaires. The same questionnaires were distributed to participants after the training sessions. We have linked the pre-training questionnaires with post-training questionnaires (to have two questionnaires for the same person). Of the 202 participants, 145 completed both pre-training and post-training questionnaires (response rate 72%), and thus were included in the analysis (Figure 1). 

Pre-training and post-training questionnaires were identical, and had the following groups or collections of questions:

- sociodemographic characteristics (sex, age, institutions, position, etc.);
- awareness of health inequalities (concept, vulnerable groups, causes, etc.);
- monitoring of health inequalities (the need for monitoring, indicators for evaluating health inequalities, actions for improving the monitoring of health inequalities, etc.);
- reduction of health inequalities (responsible institutions, measures, and principles for reducing health inequalities etc.);
- multi-sector collaboration (the need for multi-sector collaboration, factors that facilitate and retard collaboration, leading institutions, etc.).

Some extra questions regarding the evaluation of the quality of the training sessions were added to the post-training questionnaire. The majority of questions were presented as statements, and respondents were asked to evaluate them using a Likert scale (where zero indicates the lowest and 10 indicates the highest possible evaluations). The questionnaire is published at the web page of the project [13]. 

Data were computed, coded, and analyzed using the Statistical Package for the Social Sciences for Windows, Version 24.0 (SPSS Inc., IBM, Armonk, NY, United States). The distribution of investigated variables was analyzed using descriptive statistics, and results were presented as percentages (%) and absolute numbers (n). The normality distribution of the variables was evaluated by the Kolmogorov–Smirnov test. It transpired that all of the analyzed subscales had non-normal distribution. Therefore, continuous variables were expressed as median and range (minimum–maximum values), and a nonparametric (Wilcoxon) test was used in statistical analysis. Differences in results at the *p* < 0.05 level were considered statistically significant. 

As this study does not meet the criteria for biomedical research, there was no requirement for getting permission from the regional biomedical research ethics committee. The protocol and questionnaire was evaluated, and permission was granted by the Bioethics Centre at the Lithuanian University of Health Sciences (04-04-2017, BEC-VSV(M)-103). 

## 3. Results

Of 145 respondents, 128 (88.3%) were female, and 17 (11.7%) were male. The age range was from 23 to 64 years (mean 38.21 ± 10.81). Other sociodemographic characteristics of the training session respondents are presented in Table 2.

Respondents were asked to identify the general importance of health inequalities to the national public health agenda. The pre-training median was nine (minimum four; maximum 10), and the post-training median was 10 (minimum five; maximum 10) (*p* < 0.001). More detailed analysis suggested that 66 (45.5%) participants increased their levels of understanding and capabilities through these evaluations after the training interventions.

It was intended to evaluate the change of participants’ attitudes regarding the main causes of health inequalities in Lithuania. The results have revealed that the main causes of health inequalities are health-threatening behaviors when choice is limited or not possible; and health-threatening living and working conditions. These two causes have been evaluated as the most important factors before and after training sessions. Moreover, the evaluations by respondents regarding this targeted learning and knowledge being statistically significant increased (Table 3).

Unemployed, low-paid, and low-educated persons were identified as the most vulnerable groups of society in terms of health inequalities. However, the most remarkable changes were observed for the evaluation of *‘children from single-parent families’* and *‘low-educated persons’*. A total of 87 respondents (60.0%) (for children from single-parent families) and 98 (67.6%) respondents (for low-educated persons) recorded higher evaluation scores after training sessions (Table 4).

Respondents were asked to express their opinion on the effectiveness of selected measures in tackling health inequalities in Lithuania. The list of the measures that was presented to participants was developed based on health promotion principles identified in the Ottawa charter [14]. A more effective tobacco and alcohol control was identified as the most important measure. However, interventions such as *‘Build healthy public policy’*, *‘Create supportive environment’*, and *‘health education’* were also considered as very effective measures. It is noteworthy that all of the listed measures relating to understanding and capability have received more favorable evaluations after training sessions except in regard to *‘Improvement of health care services’*. The most significant changes were observed for *‘Improvement of social support system’*; 79 (54.5%) respondents thought that this measure could be more effective than they did before the training sessions (Table 5).

## 4. Discussion

The World Health Organization (WHO) Commission on Social Determinants of Health identified a set of recommendations for reducing health inequalities. One of these recommendations sets out that:

*‘Educational institutions and relevant ministries act to increase understanding of the social determinants of health among non-medical professionals and the general public (Rec 10.2)’*.

[15]

The results from our study illustrate that capacity-building seminars can have a considerable impact on both understanding and preparedness for addressing health inequalities. Our findings are in line with similar studies that evaluated the effectiveness of capacity-building seminars for an increased awareness of health inequalities [16] and other public health issues [17]. Therefore, it is expected that Norway Grants support and the completed training will have a sustainable effect and facilitate the further reduction of health inequalities in Lithuania [18].

It has been noted that this project and the Norway Grants support has generated awareness on health inequalities and the social determinants that has increased awareness and engagement not only among participants of project training sessions, but in the general population as well. However, it is agreed that raising awareness of the importance of social determinants of health and health equity among policy actors is important, but in itself, it is not enough [15]. It is important to highlight that since the Black Report was published in England in 1980 on inequalities in health [19], serious and systematic action to tackle inequalities in health remains limited and elusive in most countries. The European Review on the Social Determinants of Health and Health Divide [20], which informed the European Policy Framework and Strategy for Health and Wellbeing: Health 2020 [21], provides a wealth of information on the root causes of inequalities and effective ways to address them. Among them, equity was the cornerstone and the number one target issue of the *‘Strategy Health for All’* [22] in the early 1980s. Equity, and the needs of the ‘have nots’, also have a central place in the new Sustainable Development Agenda [23].

Over the last four decades since that earlier work, there has been a wide recognition of the importance of addressing inequalities on moral and economic grounds. However, inequalities are often perceived in narrow ways, most commonly in terms of ensuring access to health services or addressing the needs of certain vulnerable groups. The studies on the social determinants of health have significantly broadened our understanding of the causes of inequalities (social, economic, environmental, and cultural), and the upstream actions that could address them.

Health is a political choice, and it relates to the kind of society that we wish to have. It seems that the Lithuanian national health policy is moving in the right direction, and pays considerable attention to the issue of health inequalities. As mentioned earlier, the main objective of the Lithuanian Health Strategy 2014–2025 stresses the importance of creating a safer social environment and reducing health inequalities and social exclusion in the country [8]. Moreover, the priority to reduce health inequalities is highlighted in the national health policy document Action Plan for the Reduction of Health Inequalities in Lithuania 2014–2023 [24]. This action plan focuses on specific measures that could contribute to reducing differences in accessibility to health care services, gaps in health-threatening behavior, and health inequalities in general. The most recent Program for the Government of the Republic of Lithuania emphasizes the need for further actions in eliminating gaps in health and health care [25].

Our project demonstrates that awareness, learning, and engagement supports the engagement and involvement of people in all parts of government and society in dealing with the key areas of inequality and intersectoral collaboration to the agreed quality of life goals.

Identifying equity as a target is very important, but far more important is how we go about addressing inequalities. Many interventions can make inequalities worse, even when introducing well-intended policies. It is important to use both targeted and universal measures, ensuring a mix of policies. It is crucial to be clear about the gains and the costs of action and inaction. Upstream action means addressing the social determinants and taking actions to mitigate vulnerabilities. A key aspect of comprehensive action is the importance of intervening at the different levels where inequalities arise (social context, exposures, differential vulnerabilities of population groups, access to services, and differential consequences at the individual level). The experience from other countries suggests that many challenges arise in targeting to have more equal possibilities for health for all members of society [26]. One of the key challenges for tackling health inequalities is a mutual understanding and cooperation between national and local level institutions. This issue was identified in studies carried out in Lithuania [27] and Norway [28]. Therefore, the project’s capacity training sessions have tried to involve the representatives of different institutions representing national and local levels. The three key words for successful action are leadership, strategic thinking, and capacity to act in an effective and sustainable way. Applying the equity lens should become part of the culture of our organization. Awareness is essential. Active awareness is enabled through opportunities to learn and debate issues in multi-disciplinary groups and representatives from different organizations and industry or professional sectors. 

Giving a healthy start in life, for example, is one of the most formidable goals of any comprehensive policy to address equity and the social determinants of health. To do this would require opportunities and mechanisms to share an understanding and shape strategies and plans, as well as joint accountability that would involve the social, education, health, environmental, and housing sectors. Strong leadership is also essential [29]. However, still, its impact is lacking [30].

The training project represents an important investment toward creating a critical mass of decision-makers and professionals to contribute to the national effort to address inequalities in health. It is an important step on which to build. It is encouraging that the training was overall very well received. The timing is right to scale up action reaching out to other sectors and creating platforms to debate and better understand the meaning of seriously tackling and preventing inequalities in health.

This study has some methodological issues requiring explanation. First, the study has no control group. We were comparing the answers of respondents before and after trainings, and this methodological aspect could limit confidence in the results and conclusions. However, we believe that these finding provide a relevant picture of the impact of trainings on awareness about health inequalities. Second, the results were possibly limited in part by the use of evaluation techniques. We have used the self-evaluation technique. No validation was performed before the study, which brings reliability and validity into question. Finally, we distributed questionnaires immediately after training sessions. Therefore, these changes could have a short-term effect. We are planning to repeat the same study in 2019 and check the long-term effect of these trainings.

## 5. Conclusions

Addressing inequalities in health represents an imperative for all policies and strategies for health, quality of life, and sustainable development in the 21st century. The evidence on the gains and costs of action and inaction is strong and compelling. The evidence on how best to tackle or prevent the avoidable inequalities in health is robust and rich in practical applications. Strong leadership at all levels of government and strategic thinking remain crucial preconditions of success. However, making it happen depends on adequate and sustainable interprofessional and cross-sectoral capacity. Awareness of the importance of addressing inequalities and a good understanding of related concepts and approaches are essential for generating commitment, consensus, and legitimacy for change and innovation. This study reflects the value of investing in training and sensitizing decision-makers, professionals, and civil society. Such investment is critical and necessary in fulfilling the goals of the Lithuanian Health Strategy for 2014–2025. In this context, the Norwegian Grants that supported the project training activities were most valuable and timely.

## Figures and Tables

**Table 1:** Outline of training for the “Tackling of inequalities in health and health care: situation, challenges, and possibilities” sessions.

Topic
1. Health and health care inequalities: definition, causes, and vulnerable groups. Situation in Lithuania.
2. Monitoring and evaluation of health and health care inequalities.
3. Rural-–urban health inequalities: evaluation and causes.
4. Principles of health inequalities reduction. ‘Good-practice’ examples.
5. Intersectoral and inter-institutional collaboration at municipal level in tackling health inequalities.
6. Integration of tackling health inequalities in municipal agendas.
7. Rural–urban health inequalities: possibilities for reduction.
8. Tackling health inequalities in Lithuania: now or never.

**Table 2:** The main sociodemographic characteristics of the respondents in this study.

Variable	*n*	%
*Sex*		
Females	128	88.3
Males	17	11.7
*Representing institution*		
Public health bureaus	62	42.8
Administration of municipality	23	15.9
Ministry of Health	2	1.4
Institutions subordinate to the Ministry of Health	41	28.3
Other	17	11.7
*Position*		
Director/Deputy director	52	35.9
Specialist	86	59.3
Other	7	4.8
*Education degree*		
Doctor (PhD)	5	3.5
Master	94	65.3
Bachelor	42	29.2
No university degree	3	2.1

*n*—absolute number, %—percent value.

**Table 3:** The attitudes of study participants regarding leading causes of health inequalities in Lithuania.

Causes	Pre-Training	Post-Training	*p*	Change	
	Median (min. – max. ^a^)	Median (min. – max. ^a^)		Lower (*n*)	Higher (*n*)
Natural biological variations (ex. age)	7 (0–10)	7 (0–10)	0.599	58	62
Freely chosen unhealthy lifestyle (ex. smoking)	8 (1–10)	9 (1–10)	<0.001	43	79
Freely chosen healthy lifestyle (ex. healthy nutrition)	8 (0–10)	9 (0–10)	<0.001	40	72
Health-threatening behavior, when choice is limited or not possible	8 (3–10)	9 (4–10)	<0.001	21	73
Health-threatening living and working conditions	8 (4–10)	9 (2–10)	0.007	38	62
Inadequate and inaccessible health care services	9 (1–10]	8 (1–10)	0.990	55	46
Health-related social mobility, when health problems cause socioeconomic difficulties	8 (1–10]	9 (4–10)	0.074	43	60
The gap between government and needs of society members	8 (2–10)	9 (1–10)	<0.001	36	73

^a^ 0—not important, 10—very important; (min. – max.)—minimal and maximal values; n—number of respondents who changed their evaluations after the trainings: Lower—number of respondents who gave lower evaluations after trainings, Higher—number of respondents who increased their evaluations after trainings; p—probability of error based on Wilcoxon criteria.

**Table 4:** The most vulnerable groups for health inequalities reported by respondents in this study.

Group of Population	Pre-Training	Post-Training	*p*	Change	
	Median (min. – max. ^a^)	Median (min. – max. ^a^)		Lower (*n*)	Higher (*n*)
Low-income group	9 (1–10)	10 (2–10)	<0.001	29	59
Unemployed	9 (1–10)	10 (0–10)	0.051	37	54
Affect by stress and/or other environmental hazards	8 (0–10)	9 (2–10)	<0.001	33	69
Children from single-parent families	7 (0–10)	9 (2–10)	<0.001	35	87
Low-educated persons	8 (0–10)	9 (1–10)	<0.001	19	98

^a^ 0—not important, 10—very important; (min. – max.)—minimal and maximal values; n—number of respondents who changed their evaluations after the trainings; Lower—number of respondents who gave lower evaluations after training; Higher—number of respondents who increased their evaluations after trainings; p—probability of error based on Wilcoxon criteria.

**Table 5:** The evaluation of effectiveness of selective measures against health inequalities.

Measures against Health Inequalities	Pre-Training	Post-Training	*p*	Change	
	Median (min. – max. ^a^)	Median (min. – max. ^a^)		Lower (*n*)	Higher (*n*)
Build healthy public policy	10 (0–10)	10 (5–10)	0.02	29	46
Balanced economic growth	9 (0–10)	10 (4–10)	0.01	32	44
Reduction of incomes inequalities	9 (0–10)	9 (4–10)	<0.001	33	59
Improvement of social support system	8 (0–10)	9 (4–10)	<0.001	32	79
Create health-supporting environment	9 (2–10)	10 (2–10)	0.016	33	49
Reduction of poverty	9 (4–10)	10 (4–10)	0.076	40	47
Improvement of working environment	9 (4–10)	9 (3–10)	0.05	36	61
Reduction of unemployment	9 (4–10)	10 (0–10)	0.05	33	51
Strengthening activities of communities	9 (5–10)	9 (1–10)	0.231	40	49
Adult education programs	9 (3–10)	9 (5–10)	<0.001	31	71
Development of personal skills	10 (1–10)	10 (6–10)	0.188	32	45
Health education	9 (1–10)	10 (6–10)	0.019	32	56
Tobacco and alcohol control	9 (1–10)	10 (7–10)	<0.001	21	70
Improvement of nutrition, increase of physical activity, and reduction of obesity	9 (5–10)	10 (3–10)	0.001	25	55
Programs focused on positive parenthood development	9 (2–10)	10 (5–10)	<0.001	27	68
Reorient health services	9 (0–10)	9 (3–10)	0.460	46	44
Improvement of health care services	9 (5–10)	9 (2–10)	0.145	50	41
Increase of accessibility to health care services	9 (4–10SS)	9 (1–10)	0.003	61	31

^a^ 0—not important, 10—very important; (min. – max.)—minimal and maximal values; n—number of respondents who changed their evaluations after the trainings; Lower—number of respondents who gave lower evaluations after trainings; Higher—number of respondents who increased their evaluations after trainings; p—probability of error based on Wilcoxon criteria.
